# Experiences of young mothers with the uptake of Sulfadoxine-Pyrimethamine for intermittent preventive treatment of malaria in pregnancy: a cross-sectional study in the Lake endemic region, Kenya

**DOI:** 10.3389/fgwh.2024.1294893

**Published:** 2024-03-26

**Authors:** Dennis Juma Matanda, Tchaiwe Zulu, George Odwe, Oscar Okoth, Zoe Nakuya

**Affiliations:** ^1^ International Programs, Population Council—Kenya, Nairobi, Kenya; ^2^ Quality Department, Kisumu Medical and Education Trust (KMET), Kisumu, Kenya

**Keywords:** malaria in pregnancy, intermittent preventive treatment, Sulfadoxine-Pyrimethamine, young mothers, Kenya

## Abstract

**Background:**

A substantial proportion of the world population is affected by malaria with 241 million malaria cases reported globally. Intermittent Preventive Treatment in pregnancy (IPTp) with Sulfadoxine-Pyrimethamine (SP) is an effective chemotherapy but its utilisation has not been optimised. Few studies focus on young mothers and their experiences regarding the optimal uptake of IPTp-SP.

**Methods:**

The study design was cross-sectional with data derived from six focus group discussions with mothers aged 15–24 years who had a pregnancy and gave birth to a live baby within the last two years in Kisumu and Migori counties, Kenya. Inductive analysis was used to identify themes and patterns.

**Results:**

Young mothers were motivated to take IPTp-SP during pregnancy if they had prior knowledge about SP and its associated benefits and if they were knowledgeable about the consequences of malaria infection during pregnancy. Perceived side effects of SP, lack of awareness of SP as a malaria prevention therapy, lack of knowledge on the benefits of SP, dosage and frequency of uptake, poor communication by health providers towards young mothers, and inconsistent supply of SP at health facilities inhibited young mothers from attaining the recommended 3+ doses of IPTp-SP.

**Conclusions:**

There is a need for health literacy programmes that focus on increasing knowledge of IPTp-SP dosage, timing and benefits for both the young pregnant mother and her foetus. Community engagement through dialogue with mentor mothers and male partners will be an important complementary approach in establishing a support system for young women for positive health outcomes including attaining the recommended 3+ doses of IPTp-SP.

## Introduction

The World Health Organization (WHO) estimates that a substantial proportion of the world population is affected by malaria with more than 241 million malaria cases reported in 2021. Among the 241 million cases, sub-Saharan Africa (SSA) bears the heaviest burden of malaria accounting for 95% of the cases (1). Globally, an estimated 15% of maternal deaths, mainly in SSA, are attributed to malaria in pregnancy (MiP) (2). In Kenya, nearly a third of its 51 million people live in malaria-endemic zones with millions more exposed to the seasonal transmission of the disease (3). Malaria is one of the leading causes of morbidity and mortality in Kenya accounting for 31% of outpatient cases (4). Pregnant women are especially susceptible to malaria infection with over 1.8 million pregnant women in Kenya at risk (5). Malaria in pregnancy is one of the most common causes of spontaneous abortion and has a devasting impact on health outcomes for pregnant women and infants including severe maternal anaemia, low birth weight, preterm delivery and stillbirth (6).

Intermittent Preventive Treatment in pregnancy (IPTp) with Sulfadoxine-Pyrimethamine (SP) is one of the most effective and clinically proven malaria preventive chemotherapy recommended by WHO that can prevent the adverse effects of malaria on maternal and foetal outcomes (7). Despite the evidence showing that IPTp-SP intervention can significantly reduce the risk of adverse health outcomes attributable to MiP, efforts to scale-up this intervention have been weak or lacking in SSA (8–10). Though originally introduced for malaria prophylaxis and treatment for the general population, the decline in efficacy of SP for these purposes led to its re-designation as an effective drug for IPTp for pregnant women living in areas of sustained malaria transmission. Evidence shows that the provision of IPTp-SP to all pregnant women has a sizeable impact on maternal and neonatal health outcomes in malaria-endemic settings even after taking into account the SP resistance (11).

A meta-analysis on the efficacy of IPTp-SP showed that three or more doses of SP given at least one month apart were associated with higher mean birth weight, fewer cases of low birth weight and less placental malaria compared to two doses of SP (11). Drawing from this evidence, WHO updated the guidelines on IPTp in 2014 stating that all HIV-negative pregnant women in areas of moderate-to-high transmission in SSA receive at least three or more doses of SP throughout their pregnancy (6). As per the recommendations, SP should be administered by Direct Observational Therapy (DOT) with a minimum of three SP doses (three tablets as a single dose) to be administered one month apart as early as possible starting in the second trimester (from 12 weeks gestation) until delivery. The current Antenatal Care (ANC) model of a minimum of eight contacts with a health provider is an opportunity to administer the required three minimum doses of IPTp-SP during ANC visits (7).

The Kenyan government has adopted the WHO recommendations on IPTp-SP with SP recommended for all 14 malaria-endemic counties in the country—areas around Lake Victoria in Western Kenya and the coastal region of the country (12). However, since the adoption of the WHO recommendations in 2014 and the country developing the MiP strategy, IPTp-SP coverage has remained well below the national target of 80% for three doses (13). The 2020 Kenya Malaria Indicator Survey (KMIS) showed that only 22% of women aged 15–49 years with a live birth in the past two years before the survey had received three or more doses of IPTp-SP (13).

Throughout the literature on MiP, there is a tendency to generalise findings on factors associated with optimal uptake of IPTp-SP to women of reproductive age (15–49 years). This generalisation fails to capture the complex patterns and inequities in uptake of health services especially by young women who often experience severe maternal health outcomes compared to their older counterparts (14). Importantly, evidence on uptake of IPTp-SP has shown that younger women are at a greater risk of not achieving the recommended three or more doses of SP during their pregnancy (15, 16). Therefore, this study aimed to explore the experiences of young mothers around access, administration, and frequency of uptake of IPTp-SP; and factors that motivate or hinder young women from attaining optimal uptake of IPTp-SP.

## Materials and methods

### Study design

This study is part of a larger project, the Revive IPTp-SP project which aims to revitalise and support the effective delivery and uptake of SP for intermittent preventive treatment of malaria in pregnancy in Kisumu and Migori counties in Kenya. The project is addressing the low uptake of IPTp by: (i) increasing program implementers' and healthcare providers' understanding, capacity, and willingness to promote use of SP in IPTp; (ii) increasing community engagement on IPTp and promoting self-care by pregnant women; and (iii) strengthening the capacity of county government bodies to deliver and monitor IPTp-SP interventions as part of the country's devolved healthcare system. Data used for this study comes from a cross-sectional baseline survey aimed at generating evidence on women's knowledge, attitudes and practices regarding MiP prevention during their most recent pregnancy. Specifically, data for this paper was derived from six focus group discussions (FGDs) with women aged 15–24 years who had a pregnancy and gave birth to a live baby within the last two years preceding the survey.

### Study setting

The study was conducted in two malaria-endemic counties of Migori and Kisumu that have experienced low coverage of IPTp (17). The Kenyan health system is categorised into public providers, private non-profit organisations (including faith-based and mission hospitals as well as local and international NGOs), and private for-profit healthcare providers. Public healthcare is provided at primary healthcare centres and dispensaries where services for simple ailments and ANC can be accessed. Patients with conditions that cannot be handled at the primary healthcare centres are referred to clinics and hospitals. Migori and Kisumu counties have a total population of 2.27 million people (3). An estimated 52% of the population are women, half of whom are women of reproductive age (15–49 years). Despite being IPTp-focus counties, the 2014 Kenya Demographic and Health Survey (KDHS) showed that only 6% and 13% of pregnant women in Kisumu and Migori counties respectively received three or more doses of IPTp-SP (18). Recent estimates from the 2020 malaria indicator survey showed an improvement in the uptake of three or more doses of IPTp-SP in the Lake endemic zone (encompassing Kisumu, Migori and other counties near Lake Victoria) from 35% in 2015 to 49% in 2020 (13).

### Study participants

Participants in FGDs were purposively identified in the community with the help of community health volunteers (CHV) and sub-county malaria coordinators, with approximately 8–10 participants per group. The target was women aged 15–49 years who had a pregnancy and gave birth to a live baby within the last two years in Kisumu and Migori counties. This paper is based on analysis of data from FGDs with young women aged 15–24 years (3 FGDs in Kisumu and 3 FGDs in Migori).

### Data collection

Data collection took three months beginning in June to August 2021. Data was collected by trained research assistants with social science background and prior experience in conducting qualitative interviews. The research assistants attended a five-days’ workshop where they were trained in conducting research in an ethically appropriate manner. The training also involved mock interview sessions and a pre-test exercise with a similar study population. Experiences from mock interviews and pre-test were used to revise data collection tools for actual data collection.

Using a guide, each FGD was facilitated by two research assistants (one facilitator and one note-taker). The note-taker also ensured that the interview was audio recorded with the participants' consent using a digital sound recorder. The FGD guide comprised of questions on: factors that determine whether pregnant women seek care at health facilities, individual experiences regarding the kind of care received at health facilities during pregnancy, factors that determine whether pregnant women take SP/Fansidar for prevention of malaria during pregnancy, and individual experiences regarding access to SP/Fansidar for prevention of malaria during pregnancy at the health facility and community level.

### Data analysis

Audio recordings of FGDs were transcribed verbatim, typed in Microsoft Word and analysed for content using NVivo version 12 software. Interviews conducted in Kiswahili or local language (Dholuo) were directly translated and transcribed into English. An inductive analysis approach was used to identify themes and patterns across the various interview groups. First, data analysts coded the same transcript and compared codes to ensure consistency of the coding between the coders. Codes corresponding to themes and constructs were entered into the database and used to organise data for refined analysis.

Data analysis involved a team's approach to both creating data summaries and identifying themes in an effort to improve the reliability of the analysis. Data analysts independently identified topics and patterns, and then met regularly to discuss the interpretation of emerging themes and identify exemplar quotes from the transcripts that illustrated a certain theme. Intercoder reliability tests were conducted to ensure consistency in coding between coders. The intercoder reliability test produced a Kappa coefficient value of 0.80, with 0.75 indicating the minimum threshold for excellent agreement (19).

## Results

### Access, administration and frequency of uptake of IPTp-SP among young mothers

Participants were asked to share their experiences regarding access to SP, the administration method, when they started taking SP, and the frequency of receiving SP during their most recent pregnancy in the last two years. Participants' responses showed variability in terms of SP availability depending on where they lived. For example, young mothers in four out of the six sub-counties where FGDs were conducted indicated availability of SP in facilities when they visited contrary to young mothers in two sub-counties who experienced unavailability of SP due to stockouts.

*“It was available in the hospital because when I came to the hospital, I found it… I took it three times.”* FGD-R1-N, Aged 18 years

*“Personally, when I got pregnant, I went to the clinic but most of the time I never got the Fansidar. I told them how I was feeling, and in most cases, they told me ‘We do not have them…you can go and buy.’ So, I did [buy SP], but did not know how many times I should take them.”* FGD-R6-G, Aged 24 years

With regards to the method of administration, most of the participants stated that SP was offered through DOT as recommended by WHO. Nonetheless, a few of the participants shared that they were allowed to carry home SP after experiencing difficulties in swallowing or were asked to go purchase it in private pharmacies due to stockouts.

*“You know the doctor gives it to you right away and you take it there, they insist that you have to take it and so I just had to take it…I would just take it when I go to the clinic.”* FGD-R7-N, Aged 17 years

*“They give you the drug there, they don*’*t give you to go with them at home, we were only given the red ones to take home.”* FGD-R3-N, Aged 19 years

*“I vomited and I was told to take the medicine at home because they thought I took the medicine before eating.”* FGD-R7-G, Aged 19 years

On young mothers' experiences on when they started taking SP and its frequency, there was considerable variability both in terms of the stage in their pregnancy at which they started taking SP and the number of times they took SP. For example, some of the participants started taking SP when they were two months—which is earlier than the recommended starting time of second trimester; others started when they were three months—as recommended; while others started as late as seven months into their pregnancy.

*“The first time I went to the clinic, I was two months pregnant, and I was given the drugs and I swallowed them, I remember that I was given three times.”* FGD-R1-S, Aged 20 years

*“I started going to the clinic at three months, that is when they started giving me the drugs.”* FGD-R4-N, Aged 22 years

*“I started my clinic at seven months and it's when I received the drugs.”* FGD-R3-N, Aged 19 years

Differences were equally noted regarding the frequency at which SP was given with the majority reporting having received one or two doses, and a few who had received the recommended three or more doses of SP.

*“They only gave it to me once. I took all three tablets there. When I went back home, I was just okay and did my chores.”* FGD-R2-S, Aged 24 years

*“They told me I was lucky the drugs were available; they gave me 2 times but bought them once.”* FGD-R6-G, Aged 24 years

### Factors that influence young mothers' uptake of SP for malaria prevention during pregnancy

Study participants were asked to provide reasons that motivated or hindered them from taking SP during their most recent pregnancy in the last two years preceding the survey. Beginning with factors that facilitated uptake of SP, young mothers were more likely to take SP if they had prior knowledge about SP including its use in prevention of malaria during pregnancy, if the medicine was consistently available at the facility, and if there was good communication between young mothers and health providers who would respond to their questions and provide information about SP.

*“I found a doctor and asked her why she was giving me that medicine to swallow. She told me to swallow it there and then. I did exactly that then I asked her why I was taking that medicine because I didn*’*t know why. She explained to me how the medicine works and how it prevents malaria.”* FGD-R4-A, Aged 17 years

*“The doctors always talk about the medication. They tell you this is for malaria, and before you take them, they tell you to make sure you eat first and not to take them when hungry because they are strong.”* FGD-R3-G, Aged 24 years

*“I asked the doctor what this drug was for, and he told me that this drug is going to help me and prevents my baby from getting malaria.”* FGD-R6-S, Aged 20 years

Young mothers were also motivated to take SP in order to protect themselves from possible consequences of malaria such as miscarriage and death, and to protect the unborn child. This was informed by their previous experiences with episodes of malaria and awareness of the high prevalence of malaria in the context where they lived, especially during rainy seasons.

*“Pregnant women are delicate. I think it is easy for malaria to kill a pregnant woman because when you are pregnant, your baby depends on your blood. When you have malaria during pregnancy, your blood is affected. The drug can help to prevent her and the baby from getting malaria.”* FGD-R1-S, Aged 24 years

*“The reason why we take it is to prevent malaria. Because when you have severe malaria in the early stages of pregnancy, it can cause miscarriage.”* FGD-R2-N, Aged 18 years

*“In our community here, you find that there are many water bodies and sometimes you find next to the road there are just so many small holes with water inside them which acts as breeding places for mosquitoes. There is even a season for mosquitoes when they are very many, especially during the rainy season. That is what makes me go for the medicine to prevent me from being infected with malaria when I am pregnant.”* FGD-R8-A, Aged 18 years

Additionally, young mothers highlighted the influence of family support in encouraging them to take SP. Supportive family members such as mothers and partners/husbands played a significant role in ensuring that young pregnant women who were hesitant of taking SP were encouraged to take it.

*“I told my mum that I will not take them, but my mum was a nurse who use to tell me ‘You just have to take them even if you are vomiting.’ She used to encourage me, so I took them till I delivered.”* FGD-R6-N, Aged 23 years

*“When I took it, I had nausea, but my husband was supportive and encouraged me to take the drug.”* FGD-R4-N, Aged 19 years

With regards to factors that hindered young mothers from taking SP, a myriad of challenges were mentioned. The main ones revolved around challenges at the individual and service delivery levels. At the individual level, the most common hinderance to SP uptake mentioned was fear or experience of adverse/side effects. These included feeling nauseated leading to vomiting, dizziness, tiredness and headache. The mentioned side effects negatively impacted young mothers' perception of SP leading to either not taking the medication or not adhering to the treatment regimen.

*“When I went to the clinic, I got that drug. I took it with water, and I felt a headache and was tired for two days. When I went for the next visit I told them about it, the effects of that medicine. They gave me the same medicine and told me to continue taking it. They told me that it affected me because maybe I was unwell.”* FGD-R2-S, Aged 24 years

*“They do not take it [SP] because they vomit after taking it. So, the next time they go to the clinic they do not want to take it.”* FGD-R8-N, Aged 22 years

*“You feel very tired, with a sore mouth. When I took it, I felt bad for three days. So, I do not want to take it.”* FGD-R1-N, Aged 18 years

*“When I went to the hospital during pregnancy, I was given that drug, but I was afraid of taking it. When I took it, I felt dizzy and very unwell.”* FGD-R7-N, Aged 17 years

Other individual level factors that limited uptake of SP included lack of awareness among young pregnant mothers about SP and thus they could not ask for it if it was not provided; inadequate knowledge about the benefits of SP and the number of times pregnant women were required to take the drug; the fear of medication in general; and pregnant women believing that using an insecticide treated bed-net provided enough protection against malaria and therefore finding it unnecessary to take SP.

*“When I went there, I didn*’*t know about it. Then I was given the medicine then told to swallow it there and then. I did just that and I did not know because I didn*’*t ask so I was just instructed to swallow it.*” FGD-R7-A, Aged 19 years

*“Women are afraid of drugs. At times even the drugs to increase blood level. Sometimes we put them [drugs] under the pillow rather than take. The ones for malaria, I used to take the medication from the clinic, and I would feel sick. They made me vomit and lose my appetite; I will not go and get them again.”* FGD-R2-G, Aged 22 years

*“I was given some medicine, but I can*’*t remember whether they were two or one, after swallowing the medicine, I was told to go and I went. So, I didn*’*t ask because I didn*’*t know.”* FGD-R5-A, Aged 23 years

At the system level, poor communication between health providers and young pregnant mothers was a limiting factor in ensuring optimal uptake of IPTp-SP. The poor communication meant that pregnant women were only instructed to take SP without explaining what the drug was and why they were being asked to take it.

*“The doctor should tell people, anyone who comes to this clinic, that we are giving these drugs and that these drugs help in this and that. They should tell people.”* FGD-R2-S, Aged 20 years

*“I didn*’*t bother to ask any questions because the nurse who was attending to me was also pregnant and was in a bad mood. So, asking her so many questions irritated her. After taking the red and white medicine I then got injected, she told me to come back the next date of the clinic.”* FGD-R1-A, Aged 23 years

*“I was not given [SP] the second time, and they did not tell me why.*” FGD-R2-S, Aged 20 years

Discussants also reported cases of stockouts of SP in health facilities which compelled health providers to ask expectant women to purchase SP in private pharmacies. This significantly limited young mothers' access to SP given that majority of them depended on the free drugs offered by the government in public health facilities.

*“The doctor didn*’*t give it to me. Maybe the medicine wasn*’*t available that*’*s why he didn*’*t give it to me. This is because our facilities do miss drugs so that one [SP] wasn*’*t available.”* FGD-R3-S, Aged 19 years

*“Pregnant women should be given the nets and Fansidar, but nowadays they only give nets and not Fansidar.”* FGD-R3-G, Aged 24 years

## Discussion

This study explored the experiences of young mothers around access, administration, and frequency of uptake of IPTp-SP; and factors that motivate or hinder them from achieving the recommended 3+ doses of IPTp-SP. Study findings showed that young mothers were motivated to take IPTp-SP during pregnancy if they had prior knowledge about SP. Factors that inhibited young mothers from attaining the recommended 3+ doses of IPTp-SP included perceived side effects of SP, lack of awareness of SP as a malaria prevention therapy, lack of knowledge on the benefits of SP, dosage and frequency of uptake, poor communication by health providers towards young mothers, and inconsistent supply of SP at health facilities.

Findings of this study showed variability in availability of SP with some of the young mothers unable to access it due to stockouts experienced in health facilities where they lived. Literature on the availability of SP in countries affected by malaria in SSA shows that the majority of countries do indeed experience shortages in SP supply. For example, countries like Cameroon, Côte d'Ivoire, Ghana, Mozambique, Mali, Uganda, Tanzania and Kenya have experienced insufficient and inconsistent supply of SP which reduces the chances of eligible pregnant women receiving the recommended three or more doses of IPTp-SP (20–22). Nonetheless, evidence has shown that in circumstances where SP availability and supply has been stable, it has resulted in significant increase in optimal uptake of IPTp-SP (23, 24).

Some health providers did not appear to adhere to the provision of SP through the DOT approach. This was triggered by either pregnant women experiencing difficulties in swallowing SP and hence given the drug to swallow at home, or due to stockouts, pregnant women were advised to purchase SP at private pharmacies. Across SSA, studies have documented poor implementation of the DOT approach with regards to IPTp-SP (25, 26). Key among reported challenges in DOT implementation include absence of or poorly equipped DOT corners with no water or cups (20, 21); pregnant women giving excuses to avoid taking SP including preferring not to take SP on DOT if they have not eaten food (20, 27); and deliberate disregard of the DOT approach by health providers who sometimes empathise with pregnant women on the perceived adverse effects of SP and decide not to give SP on DOT (28, 29). Important to note is that in situations where health providers have adopted a consultative approach in implementing the DOT approach when delivering IPTp-SP, especially when dealing with young mothers, it is possible to convince hesitant pregnant women to take SP on DOT (30).

Considerable differences were noted with regards to the gestational age at which young mothers started taking IPTp-SP with some starting earlier than recommended (first trimester) or late into their pregnancy. The inconsistency on when to begin taking IPTp-SP coupled with other factors could have contributed to the variability observed in the frequency of uptake of IPTp-SP with fewer women attaining the recommended three or more doses. Inconsistencies in terms of the gestation age at which to begin taking IPTp-SP has been documented in a number of studies (28, 31). Health providers have specifically been identified for failing to administer SP at the onset of the second trimester as recommended. This has been attributed to health providers' poor knowledge of the IPTp-SP protocol and inadequate health education provided to pregnant women (20, 29, 32). In some instances, it has been reported that health providers were knowledgeable about the IPTp rationale, dosage and DOT but not timing of administration (30).

Looking at factors that motivated or hindered young mothers from taking IPTp-SP, study findings showed that at an individual level, prior knowledge about SP use in prevention of MiP and its associated benefits, knowledge of the consequences of malaria infection during pregnancy including miscarriage and death, and the desire to protect the unborn child motivated young mothers to take IPTp-SP. On the contrary, perceived adverse effects of SP; lack of awareness of SP as a malaria prevention therapy which meant that young mothers could not ask for it if not given; and lack of knowledge on the benefits of SP, dosage and frequency of uptake inhibited young mothers from attaining optimal uptake of IPTp-SP. Individual level factors that hinder optimal uptake of IPTp-SP especially perceptions and actual adverse effects associated with SP have been reported in several studies (33, 34). In Tanzania, pregnant women perceived SP as harmful to them and the foetus (35); in Mali, health providers empathised with pregnant women and omitted provision of SP to those who did not want to take SP due to perceived adverse effects of the drug (29).

Notably, contrary to the perceived adverse effects of SP, findings from Mozambique and Senegal showed that majority of pregnant women had no indications of perceived adverse reactions after ingesting SP (23, 36). It is also evident that women will typically accept IPTp if encouraged by health providers and if women's perceived benefits of IPTp-SP uptake outweigh their experience of the drug's adverse effects (24, 37). Other individual level factors that hinder young mothers from attaining optimal IPTp-SP uptake that have been reported in this study corroborate with what has been reported in other studies. This includes limited knowledge among pregnant women about IPTp-SP protocol and benefits of IPTp-SP (38, 39). These individual level factors are significantly important especially in reference to young mothers who may be having their first pregnancy with limited information on how to deal with the pregnancy.

At the household level, the influence of family support especially from parents and partners of young mothers played a significant role in encouraging those hesitant of taking IPTp-SP. A study in Ethiopia revealed that male partner participation in ANC increased attendance and utilisation of ANC services including IPTp. Active involvement of male partners made them more aware of the significance of maternal health care services which translated to increase in uptake of ANC services (40). The availability of family support, especially from male partners is relevant in many patriarchal African communities where women often lack independent access to finance and solely rely on husbands, partners, or heads of families to pay ANC related charges (41). Lack of autonomy or freedom to make independent decisions has also been negatively associated with uptake of ANC services including IPTp-SP as pregnant women find it difficult to access services if constrained by the requirement of seeking permission from the head of the household (25). Evidence shows that support and encouragement from partners and other family members can increase uptake of IPTp-SP (26).

Factors at the system level that motivated young mothers to take IPTp-SP included consistent supply of SP, and friendly, responsive and comprehensive communication between the health providers and young mothers. Service delivery challenges especially the poor communication skills of health providers towards young mothers, and the inconsistent supply of free SP in public health facilities reduce the likelihood of optimal uptake of IPTp-SP. These findings are consistent with studies conducted in SSA which highlight disrespectful providers as a barrier to access to ANC services including optimal uptake of IPTp (42, 43). Closely related to this study, a study on adolescents in Burkina Faso highlighted the negative health provider attitude towards young women whose pregnancies were often considered out of wedlock and thus contributed to poor uptake of health services (44).

These findings have programmatic implications as they clearly identify challenges that need to be addressed and areas to be strengthened as Kenya strives to achieve full protection of pregnant women living in malaria risk areas. Cognisant of this, there is need to increase utilisation of appropriate malaria interventions such as IPTp-SP especially among the vulnerable in the community including young mothers. The Revive IPTp project is currently addressing some of these challenges through community dialogues and supporting outreaches with the aim of reaching those who are likely not to access ANC services. There is need for countries to increase the availability of data disaggregated by key sociodemographic variables including age and other characteristics relevant to national contexts (45). Age disaggregation in particular is critical in generating evidence to inform the life-course approach to health which regards health and wellbeing as determined by the accumulation of risk and protective factors, encountered at different stages of life (46).

### Limitations

Findings from this study should be interpreted in light of several limitations. First, it was difficult to clearly delineate factors that solely make young mothers peculiarly disadvantaged in achieving the recommended three or more doses of IPTp-SP as the described factors tend to cut across all age groups. Nonetheless, young mothers are already vulnerable and thus these factors are likely to affect them more than older mothers. Second, study participants were purposively selected, and data collection limited to a few sub-counties within the larger Migori and Kisumu counties. Study findings are therefore not generalisable beyond the study sites. Third, the purposive sampling approach used to recruit women who had given birth to a live baby in the last two years preceding the survey left out women whose pregnancies were not carried to term. Consequently, findings from this study do not adequately capture perspectives of such women on IPTp-SP uptake. Nevertheless, the main aim of the study was to find out the experience of young mothers who had the opportunity to optimise IPTp-SP uptake throughout their pregnancy, which could be best accomplished by speaking to mothers who had carried their pregnancy to full term and given birth to a live baby.

## Conclusions

This study has shown that young mothers face a myriad of challenges for optimal uptake of IPTp-SP. Health literacy programmes focusing on increasing young mothers' knowledge of dosage, timing and benefits of IPTp-SP should be expanded. Community engagement through dialogue with mentor mothers and male partners will be an important complementary approach in establishing a support system for young mothers for positive health outcomes. Efforts should equally be made to address health system challenges. This includes improving health provider interpersonal communication skills to foster friendly interactions with clients especially when dealing with young mothers, addressing supply chain management challenges and ensuring a supported and motivated workforce.

## Data Availability

The raw data supporting the conclusions of this article will be made available by the authors, without undue reservation.
